# Chronic kidney-disease screening service quality: questionnaire survey research evidence from Taichung city

**DOI:** 10.1186/1472-6963-9-239

**Published:** 2009-12-19

**Authors:** Deng-Juin Lin, Ya-Hsin Li, Jar-Yuan Pai, Ing-Cheau Sheu, Robert Glen, Ming-Jen Chou, Ching-Yi Lee

**Affiliations:** 1 Institute of Medicine, Chung Shan Medical University, Taichung, Taiwan; 2 Department of Health Policy and Management, Chung Shan Medical University, Taichung, Taiwan; 3 Department of Health Policy and Management, Chung Shan Medical University, Taichung, Taiwan; Also Chung Shan Medical University Hospital, Taichung, Taiwan; 4 Center of General Education, Chung Shan Medical University, Taichung, Taiwan; 5 Department of Applied Foreign Languages, Chung Shan Medical University, Taichung, Taiwan

## Abstract

**Background:**

Chronic kidney disease (CKD) is a serious public health problem in Taiwan and the world. The most effective, affordable treatments involve early prevention/detection/intervention, requiring screening. Successfully implementing CKD programs requires good patient participation, affected by patient perceptions of screening service quality. Service quality improvements can help make such programs more successful. Thus, good tools for assessing service quality perceptions are important. Aim: to investigate using a modified SERVQUAL questionnaire in assessing patient expectations, perceptions, and loyalty towards kidney disease screening service quality.

**Method:**

1595 kidney disease screening program patients in Taichung City were requested to complete and return a modified kidney disease screening SERVQUAL questionnaire. 1187 returned them. Incomplete ones (102) were culled and 1085 were chosen as effective for use. Paired t-tests, correlation tests, ANOVA, LSD test, and factor analysis identified the characteristics and factors of service quality. The paired t-test tested expectation score and perception score gaps. A structural equation modeling system examined satisfaction-based components' relationships.

**Results:**

The effective response rate was 91.4%. Several methods verified validity. Cronbach's alpha on internal reliability was above 0.902. On patient satisfaction, expectation scores are high: 6.50 (0.82), but perception scores are significantly lower 6.14 (1.02). Older patients' perception scores are lower than younger patients'. Expectation and perception scores for patients with different types of jobs are significantly different. Patients higher on education have lower scores for expectation (r = -0.09) and perception (r = -0.26). Factor analysis identified three factors in the 22 item SERVQUAL form, which account for 80.8% of the total variance for the expectation scores and 86.9% of the total variance for the satisfaction scores. Expectation and perception score gaps in all 22 items are significant. The goodness-of-fit summary of the SEM results indicates that expectations and perceptions are positively correlated, perceptions and loyalty are positively correlated, but expectations and loyalty are not positively correlated.

**Conclusions:**

The results of this research suggest that the SERVQUAL instrument is a useful measurement tool in assessing and monitoring service quality in kidney disease screening services, enabling the staff to identify where service improvements are needed from the patients' perspectives.

## Background

Chronic kidney disease (CKD) is a five-stage progressive loss of renal function over a period of months or years. Each stage is a progression through an abnormally low and deteriorating glomerular filtration rate, which is usually determined indirectly by the creatinine level in the blood serum. When kidney disease progresses, it may lead to kidney failure and possibly require dialysis or a kidney transplant to maintain life. CKD can be caused by diabetes, high blood pressure, and other disorders. It can be detected through three simple tests of: blood pressure, urine albumin level, and serum creatinine level [1]. Early detection and treatment of CKD can help prevent patients' conditions from getting worse.

CKD afflicts people all over the world, and thus it is an urgent need for all countries to have a public health policy for dealing with it. In the U.S.A., for example, CKD is a serious public health problem, with national surveys there showing a considerably higher prevalence than appreciated previously [2,3]. According to the analysis of the National Kidney Foundation in the U.S.A., 26 million Americans have CKD and another 20 million more are at an increased risk of developing it. The American Diabetes Association (ADA) has stated that 20%-30% of individuals with diabetes develop CKD. This is in spite of the facts that the U.S.A. has good quality medical care and that CKD is one of the most preventable of the many serious complications of diabetes. According to Josef Coresh's study of data from National Health and Nutrition Examination Surveys (NHANES), 10% of Americans had chronic kidney disease between 1988 and 1994, and 13% between 1999 and 2004 [4,5]. Driving the increase is a dramatic rise in diabetes and high blood pressure. Each of these conditions can lead to chronic kidney disease.

In Taiwan, for another example, CKD is the eighth leading cause of death. The mortality rate increased from 11.39% per 100,000 population in 1990, to 20.8% per 100,000 population in 2004. The incidence of end-stage renal disease (ESRD) in Taiwan is the highest in the world according to ESRDS 2002 statistics. In that same year, the incidence of CKD in Taiwan ranked as the second highest in the world, just after Japan. Hsu (2006) rates the prevalence of CKD stages 3 to 5 in Taiwan at 6.9% [6]. Research also concludes that the high prevalence and low awareness of CKD in Taiwan show the need to advocate more strongly for CKD prevention and education for both physicians and the general populace [6].

Dialysis and kidney transplants are too costly for most people living outside the industrialized world, and too costly even for a large number of people living in industrialized countries. For these people, prevention, early detection, and intervention are the only cost-effective strategies for CKD treatment. For public health programs based on prevention, early detection, and intervention to succeed, however, the informed and active participation of the public is required. Health education programs can deal with the informed aspect the public's required participation, but not with all aspects of the active part of that. One important factor in how willingly and actively people cooperate in a public health program is their perceptions of the quality of the health program's service. Perceptions that the quality of the service is poor will result in less willing and less active participation, while perceptions that the service quality is good should result in an increase in the willingness and activeness of the participation, Thus, accurate and practical measurement tools for assessing participants' perceptions of the quality of health care services are important. Results of such assessments can be used for determining areas with perceived and/or actual poor service quality, so that the service quality and/or the perception of service quality of those areas can be addressed and improved.

### Defining service quality

Gronroos (1984) argued that there are two distinct constituents of service quality: the technical and functional [7]. In the health care field, technical quality focuses on the technical accuracy of medical diagnosis and procedures, while functional quality is the manner in which healthcare is provided. However, in the context of health care, technical quality is difficult for patients to evaluate [8], and this resulted in most patients evaluating health care on its functional aspects alone. Parasuraman defined service quality as the difference between customer expectations and customer perceptions, and when expectations are greater than perceptions, a service quality gap arises [9].

Patients' satisfaction should be interpreted carefully due to the lack of theoretical foundations on which the concept of satisfaction and measurement are based [8]. Patients are an active consumer of health-care services rather than merely passive recipients [10]. The validity and reliability of many studies of health-care consumers' satisfaction have been questioned [11].

The original PZB model identified 10 determinants of service quality. The subsequently developed SERQUAL [12] recast the 10 determinants into five components: tangibles, reliability, responsiveness, assurance, and empathy. These five components constitute a factor analysis of the 22 item scale. Measuring quality of care from the patient's perspective has been increasingly used and accepted in health care [13-15]. One study used the SERVQUAL service quality to measure the expectations and perceptions of Greek patients regarding dental health care [9,12,16,17]. Another, refined version of SERVQUAL was used to measure patient satisfaction in health services in Bangladesh [18], and found that the "tangible" factor was the most important factor in health service quality. Li and Amir also applied SERVQUAL to measure patient satisfaction with breastfeeding education and support services [19]. Cock concluded that REFERQUAL, which is derived from SERVQUAL, holds promise as a suitable tool for future evaluation of service quality within the Exercise Referral Systems (ERS) community [20].

## Method

### Patients and Institution

Taichung City is located in central Taiwan with a population of more than one million people. In order to improve chronic kidney disease (CKD) prevention, awareness, and education, the Taichung City Public Health Bureau conducted a series of 20 free CKD screenings and prevention lectures for the community population from 2006 to 2008 (Program Number PHB-SK-93-001), as part of health exams in community centers and eight district clinics of Taichung City. Participant recruitment was effected through advertising and word of mouth. Screening recipients voluntarily chose to participate, learning of the program from posters in community centers or city district clinics, or hearing of the program from someone else. The questionnaires were self-administered, with in-person help available.

The preventative aspect of the goal was to identify people with abnormal kidney functions in an early stage and to treat identified kidney diseases efficiently and effectively using a case management and follow-up model. Persons whose kidney function eGFR measurement was determined from the screening to be below 60 were contacted and encouraged to enroll in the CKD case management plan. The management plan included several medical interventions, such as health education by public health care nurses (in person or by phone interview) every three months during their treatment, case management register card records made by public health care nurses, and a survey of all cases' kidney functions after the first year of health education for the patients. The website shows how many people attended this program and the summarized results. The details of the personal health information results were emailed or mailed to the participants.

The study total obtained the results from 1595 kidney disease screens, of which 27.8% were male and 64.9% were patients aged above 50 years old. Based on the National Kidney Foundation's definition, the screening results show 30.3% of the participants were in Stage 1 (glomerular filtration rate >90 ml/min/1.73 m^2^), 57.2% were in Stage 2 (glomerular filtration rate between 60-89 ml/min/1.73 m^2^), 11.7% were in Stage 3 (glomerular filtration rate between 30-59 ml/min/1.73 m^2^), 0.7% were in Stage 4 (glomerular filtration rate between 15-29 ml/min/1.73 m^2^), and 0.1% were in Stage 5 (glomerular filtration rate,15 ml/min/1.73 m^2^).

An adapted and revised SERVQUAL questionnaire was used in the study. A total of 1595 consecutive patients who attended the kidney disease screening program in Taichung City were requested to fill out the questionnaire. A total of 1187 questionnaires were received, of which 102 were excluded due to incompleteness. A total of 1085 effective questionnaires were collected for analysis in the study. The paired t-test, a correlation test, ANOVA, and factor analysis were used to identify the characteristics and factors of quality in the kidney disease service. The paired t-test was used to test the gaps between patients' expectation scores and perception scores. In addition, a structural equation modeling system was used to examine the relationship between satisfaction-based components. Structural Equation Modeling (SEM) of patient satisfaction was done using the goodness-of-fit measuring model. The SEM approach is considered appropriate for estimating among multiple dependent and independent latent variables, and provides a better model of the complex relationships among satisfaction components.

### Instruments

SERVQUAL represents service quality as the discrepancy between a customer's expectations for a service offering and the customer's perceptions of the service received [12]. The original SERVQUAL contains 22 paired items on a Likert scale of five service-quality dimensions: tangibility, reliability, responsiveness, assurance, and empathy. The questionnaire used in this study (see Table 1) has three parts, and uses a 7 point Likert scale (strongly disagree = 1 to strongly agree = 7). The first part, the perception and expectation component, (quality gap) is composed of the 22 pair items on service quality. The second part, the loyalty component, has two items on loyalty, which rate overall satisfaction and willingness to recommend to a friend [21,22]. These loyalty items can serve as anchor items to examine the criterion-related validity of the scale [22]. The third part of the questionnaire is the patient background data component, on areas such as sex, age, job, and educational degree.

**Table 1:** Chronic Kidney Disease Screening Questionnaire

Measure	Description
Service Quality	**Part 1. Please mark a score from 1 to 7 for the following questions on your expectations and perceptions of the quality of the kidney disease screening service, (strongly disagree = 1 to strongly agree = 7).**
	1 Did the screening have up-to date equipment?
	2 Are the physical facilities in the screening visually appealing to you?
	3 Did the staff have a nice and neat appearance?
	4 Did the facilities and equipment in the screening correspond to their service?
	5 Did the screening provide its services in a timely manner?
	6 Did the staff have a sincere interest in solving the patients' problems?
	7 When patients had problems, was the staff sympathetic and reassuring?
	8 Did the staff provide services within their promised time?
	9 Did the screening insist on error-free records?
	10 Did the staff tell patients exactly when services would be performed?
	11 Did the staff provide prompt service?
	12 Was the staff always willing to help patients?
	13 Was the staff ever too busy to respond to your request?
	14 Did the staff instill confidence in you?
	15 Did the staff provide a sense of safety to you?
	16 Was the staff courteous?
	17 Did the staff have the knowledge to answer patients' questions?
	18 Did the staff give you individual attention?
	19 Did the screening have hours convenient to your needs?
	20 Did the staff give patients' personal attention?
	21 Did the staff seem to have the patients' best interests at heart?
	22 Did the staff understand the individual needs of the patients?

Loyalty	**Part 2. Please mark a score from 1 to 7 for the following questions on your attitude towards the kidney disease screening service, (strongly disagree = 1 to strongly agree = 7).**
	23 Your overall satisfaction and evaluation regarding the screening.
	24 Your willingness to recommend this screening to your friends.

Demographic Variable	25 Gender: 1. Female 2. Male
	26 Age: ______
	27 Job: 1. housekeeper 2. public servant 3. business worker 4. labor worker 5. farmer 6. student 7. service worker 8. other
	28 Highest Educational Degree: 1. primary school 2. junior high 3. senior high 4. college 5. post-graduate

### Power analysis

For a statistical power of 0.9999, the required sample size is 364.

According to the calculation of Get PS version 3.0, 2009, when α equals 0.05 in a two-tailed test, and the sample size is 329, the power is 0.9999. Prior data indicate that the difference in the response of matched pairs is normally distributed with standard deviation 1. If the true difference in the mean response of matched pairs is 0.3, we need to study 364 pairs of subjects to be able to reject the null hypothesis that this response difference is zero with probability (power) 0.9999.

### Trial Registration

This program been waived from trial registration by the Department of Health, Taichung City, Taiwan R.O.C. within Document PHB-SK-93-001.

### Reliability and Validity

#### Internal consistency reliability

The expectation and perception satisfaction scales had Cronbach's alpha coefficients > 0.902. The "item to total" correlations were all from 0.36 to 0.90.

#### Content validity

Content validity of the questionnaire was confirmed by 3 kidney specialists and 2 healthcare management specialists. Triangulation of content validity was achieved through several literature reviews on the SERVQUAL service model [12-14].

#### Construct validity

On the basis of a review of the literature, the latent construct of patient expectations and perceptions of quality was theorized to be multidimensional. Factor analysis of the survey data identified three dimensions for expected and perceived quality [23].

#### Criterion-related validity and predictive validity

**Figure 1:**
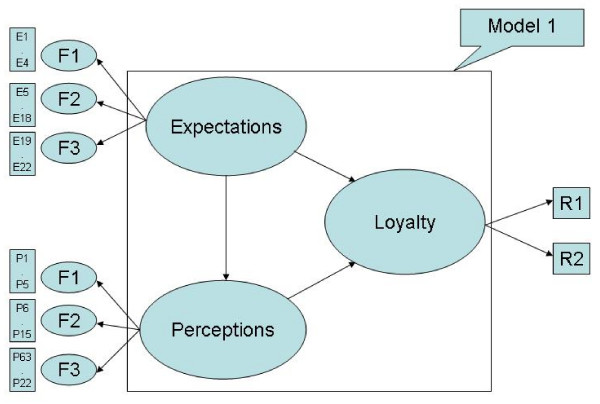
**SEM on patients' satisfaction model 1**. Indicated the initial SEM patients' satisfaction model.

**Figure 2:**
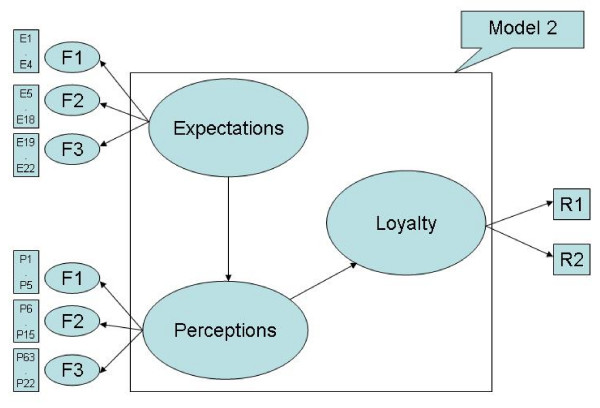
**SEM on patients' satisfaction model 2**. Indicated the final model which shows the perceptions are positively correlated with expectations. Also, loyalty is positively correlated with perceptions.

Criterion-related validity and predictive validity, shown in Figure 1 and Figure 2, indicate that the expected quality scale is correlated with the perceived quality scale, and that the perceived quality scale is correlated with the dimension of loyalty, which includes overall satisfaction and willingness to recommend to friends [22]. In addition, the goodness-of-fit indices provide model validity [24].

#### Convergent validity

Bollen's Rho coefficient equal to 0.85 and 0.91 which are greater than 0.70.

### Statistical Analysis

The software STATISTICA^® ^Version 7.1 was used for statistical analysis throughout this research. The Student t-test, a correlation test, ANOVA, and Least Significant Difference (LSD) test were used to test the average scores of expectation and perception scores with patient's characteristics. Factor analysis, which is a data-reduction technique, was used to determine the number and nature of factors of service quality that underlie our set of variables [25]. The principal axis method was used to extract all factors that had eigenvalues greater than 1, and therefore can account for a significant amount of the total variance. Scree tests were used to identify the number of factors to retain. The Paired t-test was used to test the gap between expectation scores and perception scores. Structural equation modeling was used to examine relationships among satisfaction components. The three research hypotheses of this study are as follow.

H1: Perceptions are positively correlated with expectations.

H2: Loyalty is positively correlated with perceptions.

H3: Loyalty is positively correlated with expectations.

The hypotheses were tested under the SEM using the STATISTICA^®^7.1 package. The parameters estimated were the regression coefficients in the structural equation part of the SEM. The assessment of model adequacy was based on the following goodness-of-fit criteria: normed chi-square (χ^2^/df) <3, root mean square error of approximation (RMSEA) < 0.08, population gamma index (PGI), adjusted population gamma index (APGI), goodness-of-fit (GFI), adjusted goodness-of-fit (AGFI), and Bollen's Rho >0.8 [26].

## Results

The patient's characteristics are presented in Table 2. The mean age and the standard deviation of the study population was 44.72 years old and 12.12 years, 77.97% (N = 846) of the screeners were female, and 67.1% (N = 729) of the screeners had a college degree or higher. In Table 3, the Student t-test on gender show females have significantly higher expectation levels than males. However, there is no significant difference in perception scores. Furthermore, the correlation test shows no significant relationship between age and expectation. However, other results from other studies suggest that older patients have higher perception scores. The ANOVA results show significant differences for expectation and perception scores for patients with different types of jobs. The further LSD test results reported in Table 4 and Table 5 show further details. Regarding expectations, business and labor workers have lower scores, and house keepers and farmers have higher scores. With respect to perceptions, public service and business workers have lower scores, and farmers and service workers have higher scores. The most interesting result is for the amount of education; the higher the amount, the lower the scores for both expectation (r = -0.09) and perception (r = -0.26).

**Table 2:** Patients' Characteristics

Characteristics		Range
No. of patients	1085	
Age, mean (SD)	44.72(12.12)	13-82
No. of women (%)	846(77.97%)	
Educational degree	Primary school	85
	Junior high	60
	Senior high	211
	College	576
	Post-graduate	153

**Table 3:** Satisfaction Statistical Test Results With Patient Demographics

Gender	e1-e22	Female	6.57(0.63)	t = 2.40*
		Male	6.45(0.78)	
	p1-p22	Female	6.19(0.93)	t = 0.23(NS)
		Male	6.18(0.90)	
Age	e1-e22			r = 0.05(NS)
	p1-p22			r = 0.15*
Job	e1-e22			F = 2.81*
	p1-p22			F = 4.85*
Educational degree	e1-e22			r = -0.09*
	p1-p22			r = -0.26*

Note1: * means significant at the 0.05 level

Note2: NS means not significant at the 0.05 level

**Table 4:** LSD Test On The Job Variable In Expectation

	{1} 6.616	{2} 6.547	{3} 6.341	{4} 6.259	{5} 6.824	{6} 6.414	{7} 6.653	{8} 6.429
1		0.15	0.01	0.01	0.49	0.06	0.67	0.04
2			0.04	0.03	0.36	0.20	0.22	0.18
3				0.60	0.12	0.59	0.01	0.48
4					0.08	0.34	0.01	0.27
5						0.19	0.58	0.20
6							0.06	0.91
7								0.05

**Table 5:** LSD Test On The Job Variable In Perception

	{1} 6.364	{2} 6.036	{3} 6.116	{4} 6.378	{5} 6.788	{6} 6.129	{7} 6.441	{8} 6.189
1		0.00	0.07	0.94	0.31	0.11	0.53	0.16
2			0.55	0.06	0.07	0.51	0.00	0.21
3				0.23	0.12	0.95	0.06	0.67
4					0.36	0.27	0.76	0.37
5						0.13	0.42	0.16
6							0.08	0.73
7								0.11

The patient score results are very high for expectations - 6.50(0.82), and perceptions - 6.14(1.02), as seen in Table 6. Table 6 also shows the gaps between patients' expectations and perceptions. 23 paired t tests (22 on PZB plus 1 on average) were conducted. The results show that in all dimensions patients had significantly higher scores for expectations than for perceptions.

**Table 6:** Paired t-Test Of Kidney Disease Screening Service

	Mean	SD		Mean	SD	Paired t	P value
E1	6.48	0.94	P1	5.94	1.12	14.21	0.0000
E2	6.22	1.04	P2	5.89	1.19	8.92	0.0000
E3	6.39	0.92	P3	6.17	1.01	7.10	0.0000
E4	6.56	0.80	P4	6.05	1.07	15.19	0.0000
E5	6.57	0.78	P5	6.14	1.04	13.83	0.0000
E6	6.58	0.75	P6	6.21	1.01	12.86	0.0000
E7	6.62	0.74	P7	6.26	1.02	11.75	0.0000
E8	6.57	0.75	P8	6.26	0.98	10.84	0.0000
E9	6.68	0.69	P9	6.31	0.93	13.39	0.0000
E10	6.61	0.73	P10	6.26	1.02	13.13	0.0000
E11	6.55	0.78	P11	6.25	1.00	10.01	0.0000
E12	6.62	0.73	P12	6.27	1.00	12.00	0.0000
E13	6.53	0.81	P13	6.22	1.04	10.00	0.0000
E14	6.67	0.69	P14	6.26	0.98	14.15	0.0000
E15	6.66	0.69	P15	6.25	1.01	13.41	0.0000
E16	6.60	0.73	P16	6.32	0.96	9.47	0.0000
E17	6.66	0.69	P17	6.26	0.99	13.85	0.0000
E18	6.57	0.76	P18	6.20	1.04	12.29	0.0000
E19	6.57	0.75	P19	6.25	1.05	10.56	0.0000
E20	6.45	0.90	P20	6.16	1.06	8.73	0.0000
E21	6.42	0.92	P21	6.16	1.05	7.62	0.0000
E22	6.51	0.82	P22	6.15	1.07	11.22	0.0000

Average of Expectation	6.50	0.82	Average of Perception	6.14	1.02	13.06	0.0000

N = 1085

The factor loading results of factor analysis, seen in Table 7, identify three factors in the SERVQUAL model perceived satisfaction scores (accounting for 86.91% of the total variance) and expected satisfaction scores (accounting for 80.78% of the total variance). The results in Table 8, patient expectations, Factor 1 is responsiveness, which consists of three of the original PZB model's factors: reliability, responsiveness, and assurance. Factor 2 is empathy, and Factor 3 is tangibility. However, in patient perceptions, Factor 1 is empathy, Factor 2 is tangibility, and Factor 3 is responsiveness. The eigenvalues criteria and Scree tests further confirm these 3 factors.

**Table 7:** Factor Loading Of Patient Satisfaction

	Loadings				Loadings		
			
Expected	Factor 1	Factor 2	Factor3	Perceived	Factor 1	Factor 2	Factor 3
E1			0.73	P1		0.76	
E2			0.80	P2		0.74	
E3			0.73	P3		0.67	
E4			0.63	P4		0.73	
E5	0.72			P5		0.65	
E6	0.77			P6			0.61
E7	0.77			P7			0.66
E8	0.73			P8			0.70
E9	0.83			P9			0.67
E10	0.79			P10			0.65
E11	0.65			P11			0.66
E12	0.78			P12			0.66
E13	0.67			P13			0.65
E14	0.81			P14			0.64
E15	0.80			P15			0.61
E16	0.65			P16	0.63		
E17	0.76			P17	0.70		
E18	0.63			P18	0.71		
E19		0.62		P19	0.66		
E20		0.79		P20	0.71		
E21		0.81		P21	0.75		
E22		0.74		P22	0.75		

Eigenvalue	16.1	2.1	1.87	Eigenvalue	18.00	2.21	1.39

% of Variance	70.20%	5.60%	4.98%	% of Variance	77.95%	5.18%	3.78%

**Table 8:** Questionnaire Reliability

Expectations	Cronbach's alpha	Perceptions	Cronbach's alpha
Factor 1 (E5~E18)	0.982	Factor 1 (P16~P22)	0.980
Factor 2 (E19~E22)	0.924	Factor 2 (P1 ~P5)	0.957
Factor 3 (E1 ~E4)	0.902	Factor 3 (P6~P15)	0.985

Note: Expectations 1. Responsiveness, 2. Empathy 3. Tangibles

Perceptions 1. Empathy 2. Tangibles 3. Responsiveness

Figure 1, Figure 2, and Table 9 summarize the goodness-of-fit results of the structure equation modeling, showing the directions and concepts in expectations, perceptions, and loyalty. Since Model 1 and Figure 1 do not show adequate results, Model 1 and Figure 1 have been revised into Model 2 and Figure 2. The revised model's results show adequate test results in RMSEA, PGI, APGI, GFI, AGFI, and Bollen's Rho. The value of above 0.8 is adequate and the results of the first two hypotheses are accepted. Only the χ^2^/df is unsatisfactory, being higher than the normal criteria of 3.0. Based on the SEM results, Research Hypothesis H1 (perceptions are positively correlated with expectations) and H2 (loyalty is positively correlated with perceptions) are accepted, and H3 (loyalty is positively correlated with expectations) is rejected.

**Table 9:** Goodness-Of-Fit Summary For Patient Satisfaction Models

	χ^2^	df	χ^2^/df	RMSEA	PGI	APGI	GFI	AGFI	Bollen's Rho
Model 1	7542	688	10.96	0.10	0.73	0.69	0.71	0.67	0.85
Model 2	5501	688	8.00	0.09	0.78	0.77	0.81	0.73	0.91

Note: Expectations 1. Responsiveness, 2. Empathy 3. Tangibles

Perceptions 1. Empathy 2. Tangibles 3. Responsiveness

## Discussion and Conclusion

One of the strong points of this research study is the high percentage of effective responses (1187/1595 = 74.4%), which reduces the non-response bias. This rate is just slightly lower than the 79% rate of similar research in Tso [22], but higher than the 63% response rate in Hendriks [27], the 48.8% rate in Oltedal [26], and the 25.6% rate in Bankauskaite [8]. The patients' highest expectations were on the items "Did the screening insist on error free records?" (E9) and "Did the staff instill confidence in you?" (E14), which means that patients were the most concerned about the accuracy of the screen, with respect to both the equipment used and the people who operated the equipment and administered the screens. Some mechanisms can be implemented to improve screening accuracy, for ensuring the use of well-trained medical and nursing staff, high-level technology and equipment, and the standard procedure and certification system of ISO (International Organization for Standardization) in the screening program.

The research results show generally high scores for patient expectations (6.50/7 = 92.9%) and perceptions (6.14/7 = 87.7%). In comparison, a study in India on outpatient (n = 1837) and inpatient services (n = 611) in primary health centers and district hospitals reports scores lower than those in this study, ranging from 3.63/5 = 72.6% to 3.74/5 = 74.8% [22]. In addition, the scores reported from Lin's study [15] on solo practice and group practice are also lower than those from this study, ranging from 3.73/5 = 74.6% to 4.11/5 = 82.2%. The results in this study have females with higher expectation scores than men, which is similar to results from another study which focused on asthma patients [28]. Furthermore, a study on lung cancer patients has results showing low educational level is significantly related with better patient satisfaction regarding nurse care, medical care, and other staff care, which accords with this study's results [29]. However, another research on laser-assisted in situ keratomileusis (Lasik) services showed gender and education level did not play a significant role on patients' satisfaction [30].

The results of this research suggest that the SERVQUAL instrument can be a useful measurement tool in assessing and monitoring service quality in chronic kidney disease screening service, and enabling staff to identify where improvements are needed from the patient's perspective. There were service quality gaps on all three dimensions. This means that the government-worker who administered the screenings did not meet patients' expectations, and more on-job training in areas such as etiquette are needed to provide better service. Also, positive incentives for the personnel involved in a screening to achieve higher patient satisfaction scores, and the providing of more health education and information on the screening process to those being screened to make sure their expectations are reasonable, could be effective strategies to use in the future. This study also raised a number of issues such as a need for more follow-up research on the patients from Stage 3 to Stage 5. Finally, further validation studies in various disease screening programs in Taiwan and other countries are suggested to make future cross-cultural comparisons possible.

## Limitations

This research has some limitations. One is that the questionnaires were administered during the screening process and were answered anonymously. Thus they did not include information on the severity of the participants' CKD, and were not able to be later linked to the participants who filled them out. Another limitation is that 72.2% of the participants were females, although the percentage of females in the larger general population is much less, meaning that if there is a gender-related difference in attitudes, the results are likely influenced by it. A third limitation of the study is the fact that its participants were all people who went in to a clinic or community center for a health exam, and who thus do not necessarily represent parts of the general population who for whatever reasons did not do so.

Gender selection bias is a problem for the potential representativeness of the study's results, which future research should address. As discussed in the Method section, the participants were self-recruited, and thus the recruitment/participant rate is not really applicable. In this study, gender self-selection indicated that female participants' decision to participate may be correlated with their traits, which show that females have more time to response the questionnaire than males. The traits may affect the study, making the participants a non-representative sample.

Another limitation is the 25.6% (1-74.4%) non-response bias. Furthermore, ceiling effect on the expectation and perception data also a limitation after the statistic results proved skew on data distribution. Ceiling effect can affect means, variances, reliabilities and validities of an instrument. Based on the findings of the data distribution, therefore, it implies that the effect may have direct negative consequences on patients measured by the instrument of customer satisfaction in this study.

## Competing interests

The authors declare that they have no competing interests.

## Authors' contributions

DJL was responsible for primary data collection and data clean. YHL served as a methodological consultant, assisted with data analysis and interpretation, and participated in manuscript editing. JYP was responsible for the primary study design, manuscript drafting, statistics and interpretation, and manuscript submission. ICS served as a statistics consultant, and assisted with data analysis. RG participated in the drafting and critical revision of the manuscript. MJC and CYL serve as medical consultant. All authors have read and approved this manuscript.

## Pre-publication history

The pre-publication history for this paper can be accessed here:

http://www.biomedcentral.com/1472-6963/9/239/prepub
